# Understanding Current Methods for Sampling of Aflatoxins in Corn and to Generate a Best Practice Framework

**DOI:** 10.3390/toxins14120819

**Published:** 2022-11-23

**Authors:** Rossa Donnelly, Christopher Elliott, Guangtao Zhang, Bob Baker, Julie Meneely

**Affiliations:** 1 Institute for Global Food Security, National Measurement Laboratory: Centre of Excellence in Agriculture and Food Integrity, Queen’s University Belfast, 19 Chlorine Gardens, Belfast BT9 5DL, UK; 2 School of Food Science and Technology, Faculty of Science and Technology, Thammasat University, 99 Mhu 18, Pahonyothin Road, Khong Luang 12120, Thailand; 3 The International Joint Research Center on Food Security (IJC-FOODSEC), 113 Thailand Science Park, Pahonyothin Road, Khong Luang, Pathum Thani 12120, Thailand; 4 Mars Global Food Safety Center, Yanqi Economic Development Zone, Beijing 101407, China

**Keywords:** aflatoxin, corn, representative sample, sampling procedure, sample frequency, sample size

## Abstract

Aflatoxin contamination in corn is a significant issue, posing substantial health threats to humans and animals. Aflatoxin testing protects consumer health, ensures the safe global trade of corn, and verifies compliance with legislation; however, effective sampling procedures are essential to ensure reliable results. While many sampling procedures exist, there is no evidence to indicate which is the best approach to ensure accurate detection. Using scientific and gray literature sources, this review analyzed sampling procedures to determine an optimum approach to guide the development of standard practices. Results revealed that sampling is the major source of error in the accurate assessment of aflatoxin levels in food and crucial for obtaining reliable results. To guarantee low variability and sample bias-increased sample size and sampling frequency, the use of automatic dynamic sampling techniques, adequate storage, and homogenization of aggregate samples for analysis are advised to ensure a representative sample. However, there is a lack of evidence to support this or indicate the current utilization of the reviewed procedures. Inadequate data prevented the recommendation of sample sizes or frequency for optimum practice, and thus, further research is required. There is an urgent need to make sampling procedures fit-for-purpose to obtain accurate and reliable aflatoxin measurements.

## 1. Introduction

In 2021, Mars Incorporated brought together a Food Safety Coalition of experts from industry, academia, and international organizations to drive food safety insights and standard practices at pace, starting with aflatoxins, due to the serious health threat they pose. Work was undertaken in four areas: sampling and testing, risk assessment and communication, prediction, and risk communication. This publication forms part of the work focused on sampling.

Mycotoxins are naturally occurring toxins [1] and are of major concern in the food industry worldwide, as contamination occurs regularly in food and feed commodities [2]. Approximately 25% of the global food supply is significantly contaminated [2]. Many hundreds of different mycotoxins have been identified, including aflatoxin(s) (AF(s) [3]. AFs are secondary metabolites, produced by fungi such as *Aspergillus flavus*, *Aspergillus parasiticus*, and *Aspergillus nomius*, that grow on agricultural products, including cereals, peanuts, rice, and dried fruit [4]. The four main AFs that pose a particular risk to humans, include aflatoxin B1 (AFB1), aflatoxin B2 (AFB2), aflatoxin G1 (AFG1), and aflatoxin G2 (AFG2). Among these toxins, AFB1 is considered the most harmful and prevalent in corn [2]. AF contamination occurs during crop development and maturation, and thus increases due to inadequate post-harvest conditions, including insufficient storage and drying treatments [4]. AF contamination is increasing markedly due to the impact of climate change [5]. Climate change causes variation in environmental temperatures and water activity (aw), and therefore affects fungal growth and AF production in crops [5]. *Aspergillus flavus* is highly adaptable to climate change, and consequently dominates various non-toxic fungal species [5]. Moreover, AFs are heat stable, and thus it is difficult to completely eradicate AF contamination in crops [5]. Decontamination processes including thermal processing have reduced contamination [6]. Moreover, novel-processing methods (pulsed light) have shown significant advances in AFs’ degradation [6].

AF exposure results from either direct consumption of AF-contaminated food or indirectly from food-producing animals, which have consumed AF-contaminated feed [7]. AF consumption can lead to serious health implications, as they are carcinogenic and highly toxic [8]. High AF exposure through grain consumption may cause immunosuppression, liver cirrhosis, and acute aflatoxicosis; a condition depicted by liver damage, which can possibly result in death [9]. Low levels of AF exposure over a long period of time can cause impaired growth in children [9]. Furthermore, AF consumption in animals can result in toxic effects such as chronic diseases [10], including liver damage and immunosuppression [10].

The toxic potential of AF consumption highlights the importance of testing and monitoring AF contamination in the food supply chain. Robust mycotoxin sampling procedures, coupled with the fit-for-purpose mycotoxin analysis, are essential for complying with established food safety standards and regulatory limits to confirm food is safe for trade, and human and animal consumption [11]. Many countries have established common regulations and maximum levels for AFs, and these must be supported by reliable testing data [12]. The development of effective sampling and testing methods for AF analysis is a continuing issue, as it is highly challenging to estimate the true AFs’ concentration in a batch lot due to the diverse nature of AF contamination within corn kernels [11].

The challenges associated with sampling include the fact that AFs’ concentration distributions are generally highly heterogeneous throughout a batch of bulk kernels [13]. Therefore, bulk sampling of corn may not represent the true AF contamination across an entire lot [2]. Hence, obtaining a sample that is representative of the entire batch lot is extremely difficult [2]. Variances can also occur during sampling, as minimal portions of kernels are highly contaminated, whereas the majority of the lot can be mycotoxin-free or contain negligible levels of AF contamination [13]. This can cause serious discrepancies of AF contamination being reported, including false positives and false negatives in terms of maximum residue limit (MRL) breaches, resulting in the misclassification of corn batches [2]. Therefore, these ambiguities within sampling variances threaten food safety and international trade [13].

An effective sampling procedure has a crucial role in minimizing the impact of the heterogeneous distribution of AFs in corn [11]. The key steps to obtaining an accurate measurement of AFs’ content in a lot consists of incremental sampling, sample preparation, and sample analysis via detection methods [14]. The importance of the sampling procedure is highly underestimated, but it is the most crucial component of managing AF-contaminated food safety risks [13]. Various sampling strategies have been proposed, including random and stratified [9]. Randomized sampling is more extensively utilized; however, the effects of this method in obtaining a representative sample are limited to theoretical analysis, failing to consider the heterogeneous and spatial clustering of AF contamination [9]. However, as further discussed in this review, obtaining a larger number of incremental samples at various random locations within a lot can minimize the impact of AFs’ heterogeneity, reduce sample variation, increase the reliability of results, and provide a more accurate analysis of AFs’ contamination of corn.

This review aims to provide a comprehensive overview of sampling procedures for aflatoxin analysis that have been published by governmental, non-governmental sources, and businesses (Table 1), in addition to the regulatory limits set for aflatoxins in different countries. The main themes identified from the literature reveal that official formal sampling procedures focus on sample size and frequency, thus addressing regulatory legislation and trade requirements. “Formal sampling procedures obtain samples, which are taken and analysed according to all the relevant legislation” [15]. However, informal sampling procedures focus on sampling mechanics, thus managing mycotoxin risk assessment, to ensure safe food consumption. “An Informal sampling procedure is not for enforcement purposes, but mainly a surveillance exercise to ensure food safety” [16]. Although there is an array of sampling procedures available, there is no clear indication as to which method is regarded as the best approach or has been optimized for accurate AF measurements. The overall aims of this review are to summarize the range of sample procedures identified, and critique which factors best contribute to obtaining an effective and adequate sample procedure, to produce truly representative samples. Sampling procedures have been compared and divided into informal and formal protocols. A model framework template of the standard practice has been devised for both formal and informal sampling procedures to accurately determine levels of AF contamination in corn.

## 2. Results

**Figure 1:**
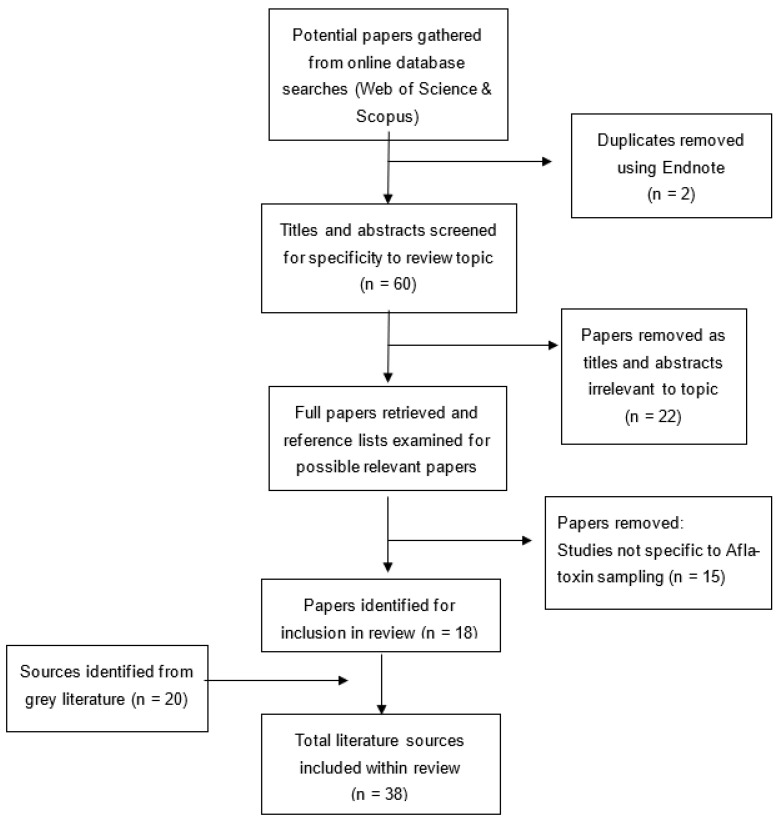
Summary of screening and critical appraisal processes.

The process for determining papers for inclusion and the collation of papers selected for use in this study is illustrated in Figure 1.

## 3. Discussion

### 3.1. Legislation and AFs’ Regulatory Limits

Due to the health threats associated with AF consumption, maximum levels of AFs have been set, to reduce these risks [12]. Moreover, the globalization of food is increasing; therefore, it is essential that there are consistent and harmonized regulations and control systems to ensure the trade of safe food [12]. More than 100 countries have established maximum levels for mycotoxin contaminants [25]; however, these limits vary greatly between countries [35]. As AFs are carcinogenic, a “no-effect” concentration or tolerable daily intake cannot be established due to the toxicity of these compounds, and thus levels in food commodities should be as low as possible [10]. Table 2 shows an overview of the regulatory limits of AFs in food adopted throughout the world. The European Commission has established the lowest maximum levels for AFB1 and total AFs (B1 + B2 + G1 + G2) in corn, conversely, America has established maximum limits only for total AFs (B1 + B2 + G1 + G2). While Europe has the lowest levels permitted for AFB1 in corn (5 µg/kg), America, China, and Nigeria have higher limits of 20 µg/kg for total AFs. The limits for most countries range from 5 to 15 µg/kg; however, Thailand and the Philippines have the highest limits permitted, i.e., 50 µg/kg for total AFs in food. It is evident from Table 2 there has been an effort to reach harmonized maximum levels between countries. This standardization will ease global trade efforts and control systems [36]. One limitation is that some countries have regulations for AFs specifying a particular food type (corn), whereas other countries have established limits for all food, therefore making it difficult to compare regulations of AFs in corn between regions.

### 3.2. AFs’ Legislative Global Impacts

The globalization of the food trade has both positive and negative impacts associated with AF contamination [38]. Countries with stricter AF limits will reject imports from countries with higher limits, resulting in extensive economic losses for certain exporting countries [39]. The EU has the strictest standard for AFs in corn, thereby reducing the global supply that can meet this standard due to higher limits in other regions (e.g., China and America). This can result in a shortage or limited supply of corn in Europe [39]. Conversely, stricter limits will ensure the export of higher quality products, including corn containing negligible concentrations of AFs [39], thus resulting in economic benefits for exporting countries [39]. Moreover, stricter limits can also improve AF-mitigation strategies and sampling techniques in exporting countries [39]. AF contamination has imposed significant economic losses in America (approximately USD 10,000 per lot annually) [38] as AF contamination led to food waste and reductions in crop price [39]. However, in developing regions, AF contamination poses a greater threat to public and animal health. The economic costs of monitoring programs, lack of political enforcement of food safety regulations, and the high reliance of corn as a staple food due to food insecurity in these regions all contribute to, and exacerbate, this food safety issue [38]. This results in significant health risks and chronic health diseases in developing regions [40,41]. The impacts associated with AF contamination indicate the need for appropriate and effective AF sampling procedures, in order to protect consumers and comply with established regulatory limits, and to ensure safe food for trade and consumption [11].

### 3.3. Sampling

The principal aim of sampling is to provide a reliable sample which represents the entire lot, i.e., “an identifiable quantity delivered at one time and assumed to have common characteristics” [42]. Various sampling procedures have been developed based on statistical parameters in association with consumer safety and producer protection [43]. Research has uncovered that only minimal corn kernels (approximately 0.1%) are highly contaminated (AF clusters), while most of the kernels are mycotoxin-free [13]. Hence, sampling must be effective for AFs, and consider their heterogeneous distribution within corn kernels [44]. Corn kernels are transported in large bulk quantities; thus, it is unrealistic to sample the entire consignment, and multiple incremental samples are withdrawn [44]. An effective sampling procedure can minimize the misclassification of lots and reduce the undesirable consequences associated with regulatory accept or reject decisions [14]. Effective sampling procedures are vitally important for developing countries for a number of reasons. Firstly, AF exposure is high in these regions and is illustrated by the high number of Aflatoxicosis cases in Africa, resulting from the consumption of contaminated corn [38] Secondly, Sub-Saharan Africa and Asia are large exporters of corn; therefore, they must ensure compliance with the regulatory limits of the countries they are exporting to [38,45]. Hence, continuous efforts are directed towards improving sampling procedures for AF analysis in foods to reduce the variability of analytical results [43].

### 3.4. Sampling Procedures

**Figure 2:**
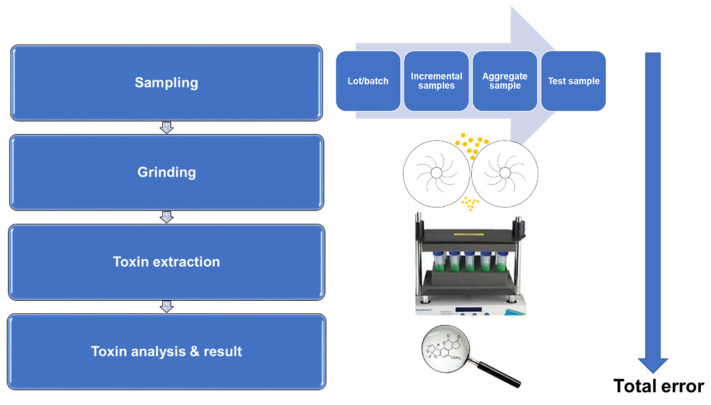
Multi-step flow diagram of AF-testing procedure.

National and international organizations have established sampling procedures for a variety of grain commodities [4,15,18,20,23,30,31,34]. Each sampling procedure contains different specifications associated with sampling; however, some procedures are more comprehensive than others. The AF-testing procedure consists of three stages: sampling, sample preparation, and analysis (Figure 2).

Each stage of the AF-testing procedure has an associated uncertainty; therefore, it is impossible to quantify the levels of AFs present in corn with 100% accuracy [2]. Furthermore, the sampling step is the most crucial step, as this is the largest contributor to error and variability [46]. To minimize variation and sampling errors and achieve a representative sample, three crucial components of the sampling procedure are highlighted within this review: the frequency and size of incremental samples in relation to the lot size, and the methods used to obtain the selected samples. A novel alternative approach, which can overcome the variance in AF sampling, is via indirect grain dust sampling [47]. This provides a fast and non-destructive sampling method for AF detection. As dust accumulates from a large quantity of grains during storage and transportation, sampling dust particles, rather than corn itself, will provide a more representative AF quantity of the entire batch [48]. Basically, the concentration of AFs present in a corn lot is indirectly related to the concentration present in dust [47]. Sampling is carried out via utilization of a rapidust system^®^, featuring a vacuum stream and a cyclone type collector [47]. Results from this sampling type [48] are promising; however, this study focused solely on wheat grain dust particles, which may not be reproducible for corn. Thus, the standardization of dust sampling is challenging to implement, and a more realistic solution is needed [47].

### 3.5. Sample Size

Many of the reviewed sampling procedures highlight that the key component of representative sampling is withdrawing sufficient quantities of many incremental samples from the lot at multiple locations [18,20,23,30], noting that this component is critical to reduce uncertainties and produce the most representative sample possible [44]. Moreover, procedures emphasize that increasing the sample size significantly reduces heterogeneous variation within a sample [15,23].

The reviewed sampling procedures include the number of incremental samples, sample frequency, the size of the required aggregate sample [15,18,20,23,30,34], or sample patterns [15,30,34]. Both the EU and FSA indicate 100 g is sufficient for all incremental samples regardless of the size of the lot or sub lots (Table 3). The FSA sample procedure is derived from the EU standard [23]. In contrast, CAC [18] and GAFTA [30] (Table 4 and Table 5) include the minimum number of incremental samples for each corresponding lot weight, which is beneficial to understand the weight of samples needed to obtain sufficient results. CAC [18] specifies lot weights from <1 to >15 Tonnes (T) (Table 4), whereas the EC [23] specifies different lot weights varying from ≤0.05 to ≥1500 T (Table 3). Therefore, the EC sampling procedure is more specific per lot weight, suggesting that following this approach could produce more representative results. Moreover, the minimum size of the aggregate sample for CAC [18] is 20 kg, compared to the range of 1–10 kg in the EC procedure [23]. Obtaining a larger aggregate sample could increase the reliability of results as it can reduce sample variation [14], thus, suggesting 20 kg is more appropriate in achieving this.

Several procedures failed to indicate exact numbers or sizes of samples per lot weight, hence limiting the sampling comparison within this review [20,34]. For example, the WFP indicates only one example of a 5000 T lot, in which 100 incremental samples should be taken weighing 220 g each [34], which is similar to EC and CAC [18,23]. However, GAFTA specifies, a lot of 5000 T will require a minimum of 50 incremental samples to be taken, withdrawing at least 50 kg per lot but a maximum 1 kg per sample [30] (Table 5). This incremental sample weight is much larger than that specified by WFP and EC, further implying a larger sample size will reduce sample variability [23,34]. GAFTA stipulates that, “as many incremental samples should be taken as physically possible” [30]; however, the International Organization for Standardization (ISO) standard of cereals and cereal products [29] stipulates for a sub lot of 500 T, 20 incremental samples (weighing between 300 and 1900 g) should be taken. This suggests a larger weight of the incremental sample should be collected, implying a greater sample weight is more significant in obtaining a representative sample. Furthermore, this stresses the inconsistencies between sample procedures. The majority of procedures acknowledge that aggregate samples with a minimum weight of 1 kg are required for analysis; however, the USDA [31] stipulates a larger minimum sample of 2.5 kg aggregate sample is sufficient. This further highlights inconsistences between the procedures. It is difficult to compare the CGC sampling procedure [20], as the specified lot sizes are “per bag” without any indication of the actual bag weight (Table 6). This is a significant flaw in the CGC protocol. The majority of corn transportation is via consignment lots by cargoes [45], not in bags. However, this illustrates that an effective sampling procedure should include both bags and lot weights as corn is transported by both means [45].

#### Sample Frequency

Advantageously, the EC, CAC, and WFP [18,23,34] procedures all provide a sampling frequency equation, to statistically estimate the time periods of sampling.

EC and CAC procedures contain the same equation:

Sampling frequency (SF) n = Lot weight × Weight of the incremental sample/Weight of the aggregate sample × Weight of individual packing

However, WFP indicates the sampling frequency using a different equation:

T (number of increment/hour) = incremental samples/sublots × Frequency.

These equations give statistical guidance of the sampling frequency, thereby reducing variation and error during sampling to create representative samples [14]. However, the method specified by the Agriculture and Horticulture Development Board [17] suggests sampling should be taken “according to the flow”, and GAFTA [30] specifies sampling points should be “carefully selected.” Each sampler’s judgment of the sampling flow will of course be different, therefore increasing the margin for error and bias during sampling, and reducing the accuracy of the overall AF detection [14]. Therefore, using the sampling frequency equation may be a better approach to reduce error and increase the reliability of results [14]. A limitation within the GAFTA [30] procedure includes the description of “uniform systematic sampling”, as systematic sampling achieves a representative sample of the entire lot [30]. However, they fail to provide an equation or guidance on obtaining the sampling frequency. The remaining procedures fail to indicate sample frequency, thus limiting comparison within this review. An advantageous feature in the GAFTA [30] procedure is the guidance of the “sampling point.” This indicates if samples are drawn outside natural daylight, there must be adequate light exposure for sampling to take place, such as artificial lighting. This was the only procedure to mention this important aspect of sampling. Moreover, GAFTA is the only procedure that briefly mentions the sampling size of grains intended for animal feed; this is particularly interesting [30], as corn is commonly used for animal feed, contributing to 95% of all animal feed in America [33].

The procedures mentioned above focus on the number of incremental samples and sampling frequency. However, USDA and GIPSA (2020) focus on providing protocols of sampling patterns using infographics, indicating multiple positions from which various samples should be taken from the lot. These include hopper cars, trucks, box cars, etc. [31]. However, these sampling patterns take fewer incremental samples than those discussed previously (8–10 per lot), further emphasizing the inconsistencies between procedures. However, these procedures are more cost-effective and less time consuming than regulatory procedures. Moreover, pattern-sampling protocols may be easier to follow, creating harmonized, systematic random sampling [14]. Most importantly, following the patterns may produce more representative samples, allowing for more accurate AF detection [9]. That said, some patterns may cause inaccurate samples to be drawn, as several sample patterns exclude the middle of the lot, and only obtain samples from the outside, thus failing to achieve a representative sample [9]. A further difference in the USDA guidance is that one incremental sample is combined with other sample(s) to create an aggregate sample, using an acceptable sampling ratio. For example, 1250 g from one carrier is added to 1250 g from another carrier, to form a 2.5 kg aggregate sample [31]. This, however, will not produce a representative sample of the entire lot, and a higher number of incremental samples are needed to increase the reliability of the sample and determine the true AF contamination [14].

The FAO and USDA emphasize the sampling procedure must allow for a high percentage of non-contaminated kernels and a low percentage of contaminated kernels [4,31]. This is extremely important in obtaining a representative sample, as it has been noted that this is a common error in sampling procedures [31]. The only feasible way to ensure this is by increasing sample frequency [44]. Statistically, the higher the number of samples taken from a lot, the higher the probability that grains containing AFs will be sampled [14]. This further highlights how sample size and frequency play a crucial role in producing an effective sampling plan for AF detection.

### 3.6. Methods and Equipment for Obtaining Incremental Samples

From the reviewed procedures, it is apparent that dynamic sampling should be performed for the optimum, representative sampling result. A large number of incremental samples collected via dynamic sampling from a moving stream provide a high possibility of any individual kernel being chosen from the entire lot [45,49]. Static sampling is challenging due to storage conditions, as moisture can drain from grains, causing variation in corn layers [50]. Hence, static sampling may only represent the part of the consignment that is assessed and will be less representative of the lot [50]. If static sampling is adopted, it is advised that the equipment must reach the entire depth of the lot [45]. CAC, ISO, ADHB, and CCG and FAO highlight how important dynamic sampling is to ensure a representative sample; therefore, it is most common and appropriate to sample corn during unloading/loading [17,18,20,26,29].

Moreover, CGC specifies sampling for both static and dynamic lots. This is unique and more beneficial for an effective sampling procedure to consist of both sampling strategies [20]. It provides a more practical approach, as both sampling strategies are performed. The CGC procedure gives in depth details of sampling static lots (grain stored in bags or totes), which are beneficial for companies who receive corn in this packaging type [20]. The static procedure is very thorough, indicating measures to avoid contamination, such as details on how to physically stroke sample sacks, preventing bursting of the sack. However, GAFTA specifies that samples should be taken from the top, middle, and bottom of each sack, to obtain a representative sample, which is not considered in the CGC procedure [30]. To achieve this, “increment samples shall be drawn uniformly, by a piercing spear from the top, middle and bottom of each bag. If it is not possible to draw a sample by spear efficiently, then the original bags may be opened to sample by hand scoop” [30].

The FAO suggests the use of automatic sampling equipment to be the most acceptable way to obtain representative samples, as it reduces human bias and removes the product from flow at regular intervals [26]. CAC, GCG, and ADHB support this, and recommend automatic sampling equipment including crosscut samplers, automatic cross-stream diverter-type sampling devices, and automatic sample buckets, respectively [17,18,20]. Using any of these options will be less time consuming and eliminate sample bias, thus ensuring random samples are collected to produce representative results. Moreover, ADHB highlights that the bucket samples are in agreement with results from standard practice recommendations [17].

**Figure 3:**
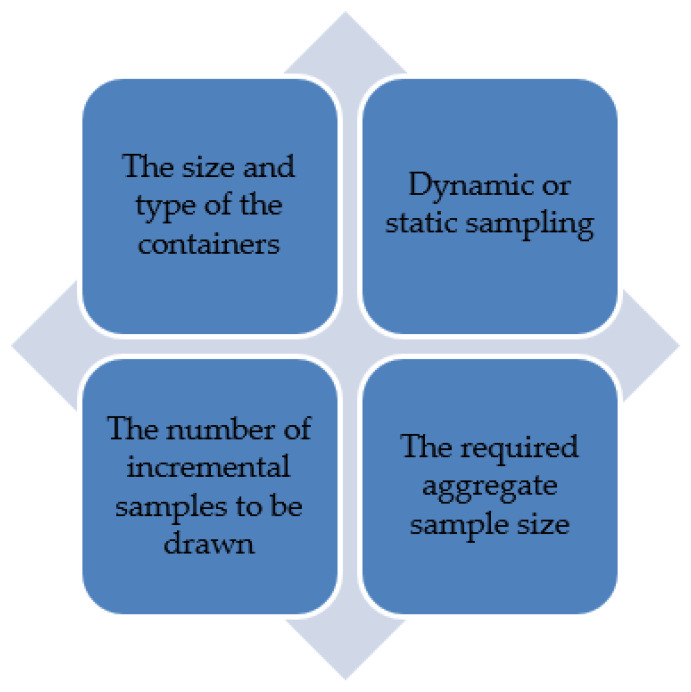
The main factors to be considered when sampling grain for mycotoxins.

Many of the reviewed procedures highlight the use of probe/triers, only if automatic sampling is not available [18,20,26,30,31], and include descriptive instructions on their use. In particular, the CGC procedure contains thorough instructions on probe/triers, thus creating comprehensive instructions for the sampler to follow, and reducing sampling mistakes [20]. Nobbe Triers are suitable for sampling free flowing products such as corn, thus reaching the center of most containers and reducing the risk of contamination [20]. However, this equipment can only sample horizontally. The double sleeve trier can sample both horizontally and vertically, but this trier poses a risk of contamination [20]. GAFTA and CGC also suggest manual handheld sampling, using a scoop [20,30]. However, this is laborious, requiring 2–3 people, and increases bias and variation within the sample [14]. Therefore, this demonstrates the use of automatic sampling equipment is the best approach, as it eliminates bias and produces more accurate results than manual sampling. To summarize, the key factors that should be considered when sampling grain for mycotoxins are outlined in Figure 3.

### 3.7. Sample Storage

Several publications have indicated the importance of suitable storage containers for incremental samples [15,18,30,31,33,37]. Sample storage is critical to reduce further contamination of corn and preserve the original characteristics of samples [15,30]. The majority of procedures indicate that samples should be stored in fully sealed, clean opaque containers, with reduced sunlight exposure [15,18,30,31,37] as a warm climate could worsen AF contamination [50]. However, the FDA recommends samples should be frozen for extended storage to avoid spoilage or mold growth [37]. ADHB provides comprehensive instructions on monitoring the temperature (best below 15 °C) and moisture content (below 14.5%) of stored samples, as grains are still a living crop during storage and are susceptible to mold growth [17]; therefore, monitoring both factors will indicate any signs which threaten corn quality.

### 3.8. Sample Preparation

Sampling strategy is only one factor that plays into the variability of test results. Sample preparation and the choice of test method all contribute to this variability and need to be taken into context when choosing a sampling method. Sample preparation is an important factor in sampling, as this step will reduce variation and the impact of heterogeneous distribution of AFs in corn, thus increasing the suitability of analytical samples for instrumental analysis [11]. One of the main factors associated with AF determination which is often overlooked is how samples are homogenized. Moreover, as the test samples are combined composite samples from the lot, this suggests sampling provides a mass average level of AFs detected from the lot, not a measure of the worst case. However, this is appropriate for AF detection in corn, as corn is often ground and mixed with other foodstuffs prior to consumption; hence, the results from the mass average AF contamination in corn are deemed suitable for this commodity.

The preparation of the composite sample is the last step in sampling prior to the mycotoxin analysis. It is often overlooked, although it is a vital component in the sampling procedure [44]. The procedures highlight the optimum equipment for comminution is a subsample mill, as this will create a homogenous and uniform test sample [4,30,37,51]. A mill can grind corn kernels without generating heat or causing a change in the moisture content [30]. Inadequate grinding creates false negatives, as smaller particles migrate to the bottom of the sample container when the sample is handled [37]. To minimize this, it is essential to thoroughly mix the sample with a high degree of comminution [52] to open and distribute the toxin throughout the particles [37]. This will ensure uniformity and reduce variability in sample preparation. The sufficient comminution of kernels will ensure the sample is representative of the bulk contamination.

### 3.9. Informal and Formal Sampling Procedure

The main differences between the sampling procedures resulted in the categorization of formal and informal guidelines (Table 7). Formal sampling procedures include: EC, FSA, GAFTA, and WFP [15,23,30,34]. These procedures mainly focus on incremental sample numbers, sample size, and frequency. This suggests that these formal guidelines mainly address regulatory requirements for trade and due diligence. However, informal procedures including USDA, ADHB, FAO, and CGC [4,17,20,31] focus on sampling mechanics and equipment used for sampling, including a lower number of incremental samples than formal guidelines. This suggests that informal procedures focus on consumer protection and mitigation strategies, due to the practicality of these procedures to ensure food safety [16]. Formal sampling procedures are more expensive and time consuming, although the utilization of sampling frequency equations will likely produce a more representative sample than obtaining samples randomly from flow [14]. However, further research is needed to support this. CAC includes common features from both formal and informal procedures, demonstrating this sample procedure may be optimum as it recommends sample size, frequency, sample method, and equipment [18]. Although the CAC sampling procedure has many advantages such as providing a sampling plan including the minimum and maximum sample size, the CAC fails to provide a limit of AFs in corn or other cereals, which is a limitation within the CAC sampling procedure. Developing countries have a limited mycotoxin sampling budget [52]. Hence, the risk between sampling cost and effective monitoring of AFs in commodities is difficult, posing a further challenge to reduce AF consumption in developing countries [52]. Therefore, an optimum AF sampling procedure for developing countries is one that must be cost-effective but will also produce accurate results and demonstrate compliance [52]. This implies an informal sampling procedure is optimal for developing countries.

#### Recommendations for Good Sampling Practice and Future Considerations

**Figure 4:**
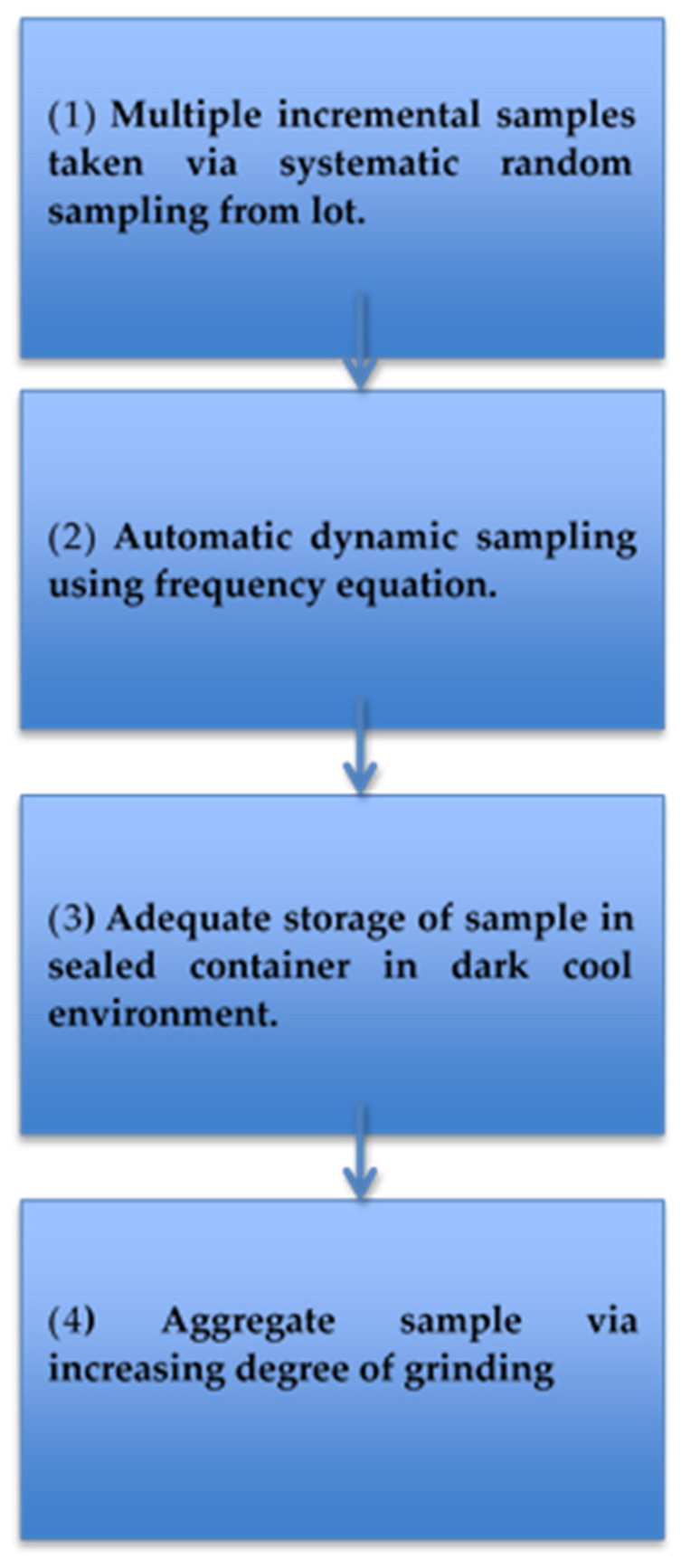
Infographic of the best approach to obtain representative samples.

All procedures highlight that the heterogeneous distribution of AFs is the main challenge associated with producing an effective sampling procedure. To overcome this challenge, key components of sampling have been recommended to ensure optimum results (Table 8 and Figure 4) and minimize the variability and uncertainties surrounding AF detection.

Despite the lack of evidence to support the effectiveness of these practices, they are deemed the best to reduce sample variation and error. The literature fails to indicate the current utilization of the reviewed procedures, or whether using these sampling procedures truly provides representative samples. This suggests the sample procedures reviewed are highly theoretical, and thus poses the following question: are these sampling procedures feasible in reality? A lack of evidence suggests they are too expensive and time consuming for companies to adhere to. Therefore, further research is needed via testing to investigate the reliability of the current sampling procedures to produce representative samples. This review could not recommend definite sample sizes or sampling frequency for standard practice due to the lack of evidence available and inconsistencies existing between sampling procedures. This highlights that further research is essential to determine optimum sampling factors. Additionally, there is a need to research alternative strategies for optimization of calculating the mass average AF concentration from composite samples taken from a lot. This will improve the reliability and accuracy of the average AF detection from composite samples. A suggested method could include testing the corn fines at the base of the transport vehicle, as these are concentration points and can be representative of the entire load. A further limitation within this review is that many developing regions including Africa do not have a mycotoxin sampling protocol to follow; therefore, effective sampling protocols should be established and implemented for these countries. Sampling protocols from India, Asia, Australia, or New Zealand are not accessible, which further limits comparison between protocols. China follows general food sampling standards from 1985, indicating the need for revised, and improved sampling procedures to produce appropriate sampling protocols for current challenges in AF sampling today. A few of the sampling procedures are also outdated, including FAO and EC [4,23]. However, the FAO provides a mycotoxin sampling tool to support the design of an effective sampling procedure for all mycotoxin and commodity combinations, which can be accessed via http://www.fstools.org/mycotoxins (accessed on 18 February 2022) [50]. This sampling tool has two main features, including evaluating the performance of a specific sampling plan and concluding the most applicable sampling plan for the type of mycotoxin (AF), to diminish the misclassification of lot acceptance or rejection. The mycotoxin sampling tool is user friendly, and a step-by-step guide is available to support its use. The tool consists of five stages (instructions, edit plans, chart results, results, plan summary, and export to software) in creating an appropriate mycotoxin sampling tool [50]. It offers a range of parameters that can be altered according to the needs of the user, including: mycotoxin type, commodity, sample size and amount, kernel count per kg, regulatory limit, analytical variance, and accept/reject limits. Altering the above sampling plan design parameters will ultimately improve the performance of the sampling plan for each user’s objective. This tool allows the determination of sampling, sample preparation, and analytical variances, thus enabling the design of an appropriate sampling plan for AFs in corn that should be considered for future sampling plans [50].

Table 9 includes a table of key principles to consider when choosing a “fit for purpose” sampling method, as this will aid developing countries in adopting an AF sampling strategy.

## 4. Conclusions

In order to ensure the safe global trade of corn, adhere to legislation, and protect consumers from AF-contaminated corn, evaluating and producing effective sampling procedures are of paramount importance. This review revealed sampling is the major source of error in AF testing, and it is the most important factor for obtaining reliable results. The most precise detection methods will not produce reliable results if the sample procedure is not representative and adequately homogenized. Insufficient sampling procedures can lead to false negatives and false positives, and result in high economic impacts and health risks. Reducing the variability within AF sampling will reduce the misclassifications of lots and is therefore the most critical factor to minimize the overall sampling error. An effective sampling procedure must have low variability and sample bias; this can be accomplished by increasing sample size and frequency, automatic dynamic sampling techniques, adequate storage, and ensuring the complete homogenization of aggregate samples for the analysis. Incorporating the recommended principles as outlined in Table 9 into an AF sampling procedure will help to ensure a representative sample is obtained. However, further research is needed to support these findings, and to obtain a fit-for-purpose sampling procedure for accurate and reliable AF detection.

## 5. Materials and Methods

### 5.1. Literature Search

The Campbell Methods Guide was followed for this review [53]. Gray literature sources and a range of peer review literature searches were examined to address the main guidelines and factors considered within AF sampling procedures for corn. Non-peer reviewed sources from the gray literature were obtained via Google search engine, to identify relevant governmental, non-governmental sources, and business publications, reports, guidelines, and standards on mycotoxin sampling procedures. Specific gray literature sources included:

- European Commission
- European Food Safety Authority
- Food and Agriculture Organization of the United Nations
- Food and Agriculture Organization
- The Codex Alimentarius Commission
- The Grain and Trade Association
- The Food Standards Agency
- The World Food Programme
- United States Department of Agriculture
- United States Food and Drug Administration

Peer-reviewed sources were obtained by conducting searches on the electronic databases Web of Science and Scopus. In order to conduct these searches, breaking down and assessing the project title identified the key words and terms. Subsequently, the key words used were (Aflatoxin* OR Aspergillis*) AND (*Corn OR *Maize) AND (Sampling* OR Sampling procedure OR Sampling procedure * protocol* OR guideline* OR standard*).

Using these electronic databases allowed for efficient identification and collection of a variety of papers from an array of scientific journals and facilitated the application of the selected inclusion and exclusion criteria. Further studies were also identified through references to various publications in the retrieved papers. After available databases were searched and relevant results returned, the online bibliographic management tool ‘Endnote’ was employed to remove any duplicate papers present.

### 5.2. Eligibility Criteria

To ensure only the relevant literature was included in the review, inclusion and exclusion criteria were determined. Inclusion criteria included literature in the English language. The inclusion of papers only in English ensured adequate understanding of the text. The exclusion criteria excluded literature prior to 1990; this allowed exploration of the literature from a wide range of information. Additionally, to assess the eligibility of the literature obtained, the literature was screened by reading titles and abstracts for key words to ensure relevance. If the literature referred to the mycotoxin, aflatoxin, corn, contaminants, sampling guidelines, sampling methods, or sample preparation, it was deemed relevant. Any papers containing peanuts or other food stuffs containing mycotoxins besides corn, or mycotoxins such as deoxynivalenol, fumonisins, zearalenone, and ochratoxin, were excluded.

### 5.3. Critical Appraisal

Critical appraisal of the publications ensured that only relevant high-quality studies were included in the review, and low-quality studies excluded. To be included in the review, papers had to adequately answer the following screening questions:

1. Does the citation indicate publication within the time period specified?
2. Is the title and abstract in English?
3. What are the main components of the sampling procedure?
4. What are the main findings of the paper?
5. Strengths?
6. Limitations?
7. Is the sampling procedure similar to others?

## Figures and Tables

**Table 1:** Summary of gray literature from Google engine.

Source	Material Accessed
ADHB	AHDB Cereals & Oilseeds is a division of the Agriculture and Horticulture Development Board (AHDB) [17].
Codex Alimentarius Commission (CAC)	Codex Alimentarius international food standards, General standard for contaminants and toxins in food and feed. CXS 193-1995. Adopted in 1995 [18]. Codex Alimentarius Procedural Manual [19].
Canadian Grain Commission (CGC)	Systems Handbook and Approval Guide (CGC; 2015) [20].
Canadian Food Inspection Agency (CFIA)	Government of Canada. Section 1—RG-8 Regulatory Guidance: Contaminants in Feed [21].
Department for Environment, Food & Rural Affairs (DEFRA)	Guidance on the organisation of informal food authenticity surveys [16].
European Union	Commission Regulation (EC) No 466/2001 [22]. Commission Regulation (EC) No 401/2006 [23].
Food and Agricultural Materials Inspection Centre (FAMIC)	Aflatoxin Regulations [24]
Food and Agriculture Organization of the United Nations/World Health Organization (FAO/WHO)	Report of an FAO technical consultation Rome, 3–6 May 1993. Sampling plan for aflatoxin analysis in peanuts and corn. (FAO 1993) [4]. Worldwide regulations for mycotoxins in food and feed [25].
Food and Drug Administration (US)	Food And Drug Administration Office of Regulatory Affairs. Mycotoxin Analysis. ORA Laboratory Manual Volume IV Section 7 [26].
Food Standards Agency	Mycotoxins Sampling Guide (2016) [15]
Food Standards Australia New Zealand (FSANZ)	Australia New Zealand Food Standards Code, 2017, Schedule 19, Maximum levels of contaminants and natural toxicants [27].
Food Safety and Standards Authority of India (FSSAI)	Food safety and standard authority of India. Manual of Methods Mycotoxins [28]
International Organization for Standardization (ISO)	ISO-24333: Cereal and cereal products—sampling. Geneva (Switzerland): ISO; 2009 [29]
The Grain and Feed Trade Association (GAFTA)	Sampling rules no. 124. Sampling, Analysis Instructions, Methods Of Analysis and Certification [30].
United States Department of Agriculture (USDA)	Grain Inspection Handbook. Grain Inspection Packers and Stockyards [31] Foreign Agricultural Service, Gain Report No. CH18026 China’s Maximum Levels for Mycotoxins in Foods [32] Feed Grains Sector at a Glance [33].
World Food Programme (WFP)	SOP for sampling and testing for Aflatoxin [34].
World Health Organization (WHO)	Mycotoxins [1]

**Table 2:** Overview of different regions’ regulatory limits of AFB1 and total AFs in food. Those highlighted in bold represent the aflatoxin limits in corn only. X = not available.

Organization	Country	AFB1 (µg/kg) (Food)	Total AFs (B1 + B2 + G1 + G2)	Food Type	References
European Union	EU	5.0	10.0	Corn	Commission Regulation (EC) No 466/2001) [22]
Food and Drug Administration	USA	X	20	Corn	United States Food and Drug Administration USDA, GIPSA (1998) [37]
	China	20	X	All food	USDA, 2018 [32]
	Africa	5	10	All food	Miklos et al., 2020 [12]
	Japan	10	10	All food	FAMIC, 2011 [24]
	Canada	X	15	All food	Canadian Food Inspection Agency, (CFIA), 2017 [21]
	Nigeria	20	X	All food	Miklos et al., 2020 [12]
	India	X	10–15	All food	Food Safety and Standards Authority of India (2020) [28]
	Australia, New Zealand	X	15	All food	Food Standards Australia New Zealand (FSANZ) (2017) [27]
ASEAN (Association of Southeast Asian Nations)	Malaysia	0.1	5–35	All food	Miklos et al., 2020 [12]
	Philippines	10	10–50		
	Singapore	0.1–5	5		
	Thailand	X	15–50		
	Vietnam	0.1–12	4–15		

**Table 3:** EU sampling plan for analysis of AFs in corn kernels by [15] and [23].

Lot Weight (Tonnes)	Weight or Number of Sub Lots	No. of Incremental Samples	Aggregate Sample Weight (kg)
≥1500	500 tonnes	100	10
>300 and <1500	3 Sub lots	100	10
≥50 and ≤300	100 tonnes	100	10
>20 and ≤50	-	100	10
>10 and ≤20	-	60	6
>3 and ≤10	-	40	4
>1 and ≤3	-	20	2
>0.5 and ≤1.0	-	10	1
>0.05 and ≤0.5	-	5	1
≤0.05	-	3	1

**Table 4:** Codex Alimentarius Commission [18] sampling plan for analysis of AFs in corn kernels.

Lot Weight (Tonnes)	Minimum Number of Incremental Samples	Minimum Size of Incremental Sample (kg)	Minimum Size of Aggregate Sample (kg)
≥15	100	0.2	20
>10 and <15	75	0.267	20
≥5 and ≤10	50	0.4	20
>1 and ≤5	25	0.8	20
≤ 1	10	2	20

**Table 5:** The Grain and Feed Trade Association [30] sampling plan for analysis of AFs in corn kernels.

Consignment Size (Tonnes)	Lot Size (Tonnes)	Number of Increments per Lot	Minimum Bulk Aggregate Sample per Lot (kg)	Maximum Weight of Increments (kg)
>25,000	500	Minimum 20	20	1
10,001–25,000	1000	Min 30	30	1
5001–10,000	2500	Min 40	40	1
0–5000	5000	Min 50	50	1

**Table 6:** Canadian Grain Commission [20] sampling plan for AF analysis of corn kernels.

Lot Size	Minimum Number of Incremental Samples
1–20 bags	All bags must be sampled
21–1000 bags	6% of all bags in lot, minimum of 20 samples randomly selected
>1000 bags	3% of all bags in lot, minimum of 20 samples randomly selected

**Table 7:** Similarities and differences between formal and informal sampling procedures.

Informal Sampling Procedure	Formal Sampling Procedure
Addresses regulatory requirements	Addresses consumer protection and mitigation strategies
Addresses number of incremental samples	Infographics and sample patterns
Much higher number of incremental samples (100)	Lower number of incremental samples (10–20)
Sample frequency determined	Random intervals depending on flow
Limited indication of static or dynamic sampling	Recommends dynamic sampling to obtain a representative sample
Limited information on sampling equipment	In-detail description on how to use sampling equipment
Indication of sample storage	Indication of sample storage
Highlights importance of homogenous ground aggregate sample	Highlights importance of homogenous ground aggregate sample
Expensive and time consuming	Less time consuming, and cost-effective

**Table 8:** Main conclusions drawn from the review to obtain “best practice”.

Main Factors Ensuring Good Practice to Minimize AFs’ Variation within Sampling
Increase number of incremental samples withdrawn.
Large incremental sample weight should be taken to produce a representative sample and reduce sample variation.
Multiple incremental samples should be drawn from multiple locations throughout the lot.
Random systematic sampling using frequency equation.
Dynamic sampling is recommended as the best approach to reduce variation and bias.
Automatic sampling equipment is recommended over manual equipment.
The aggregate sample should be fully comminuted by increasing the degree of grinding.
Incremental samples should be stored in a sealed container in a dark and cool environment.
Sampling procedure must allow for a high percentage of non-contaminated kernels and a low percent of contaminated kernels.

**Table 9:** Table highlighting key considerations for adopting AF sampling strategies.

Recommended	Not Recommended
Obtain numerous samples from random points of a lot	Obtain limited or biased samples from the same area of a lot
Obtain samples using sample frequency equation	Obtain samples randomly from flow via samplers judgment
Dynamic sampling: sample periodically from a moving stream	Static sampling
Utilize automatic sampling equipment	Use manual hand scoop (biased)
Store samples in fully sealed, clean, opaque container	Store samples in direct sunlight
Combine samples to obtain a composite sample from every part of a load	Combine samples from alike areas/points of a load
Homogenize samples fully using mill	Grind corn samples inadequately

## Data Availability

The data presented in this study are available in this article.
